# Epistemic Injustice and Illness

**DOI:** 10.1111/japp.12172

**Published:** 2016-02-08

**Authors:** Ian James Kidd, Havi Carel

**Affiliations:** ^1^ Department of PhilosophyUniversity of NottinghamUniversity ParkNottinghamNG7 2RDUK; ^2^ Department of PhilosophyUniversity of BristolCotham HouseCothamBS6 6JLUK

## Abstract

This article analyses the phenomenon of epistemic injustice within contemporary healthcare. We begin by detailing the persistent complaints patients make about their testimonial frustration and hermeneutical marginalization, and the negative impact this has on their care. We offer an epistemic analysis of this problem using Miranda Fricker's account of epistemic injustice. We detail two types of epistemic injustice, testimonial and hermeneutical, and identify the negative stereotypes and structural features of modern healthcare practices that generate them. We claim that these stereotypes and structural features render ill persons especially vulnerable to these two types of epistemic injustice. We end by proposing five avenues for further work on epistemic injustice in healthcare.

Without the narrative acts of telling and being heard, the patient cannot convey to anyone else – or to self – what he or she is going through. More radically and perhaps equally true, without these narrative acts, the patient cannot himself or herself grasp what the events of illness mean.^1^

## 1 Introduction

A study published in 1984 found that the average amount of time between a patient beginning to speak and the doctor's first interruption was eighteen seconds. Of seventy‐four office visits recorded, only in seventeen (23%) was the patient allowed to complete his or her opening statement of concerns. The authors concluded that this premature interruption of patients resulted in a loss of relevant information.^2^ We suggest that this finding is characteristic of a certain epistemic stance that tacitly incorporates presumptions about the capacities of patients to provide relevant information in healthcare contexts, and which is both epistemically unjustified and epistemically unjust.

We thus propose, in this article, to examine epistemic injustice within healthcare in the developed world. We focus on this type of healthcare in order to avoid making generalisations about other types of healthcare and in order to describe the problem in its simpler form. We note, however, that the issue described here may be prevalent elsewhere and intersect with additional problems existing in other healthcare systems.

Two broad forms of epistemic complaints reliably arise within contemporary healthcare practice. The first are what one might call *patient complaints*, those made by ill persons and especially by those with prolonged and involved experience of modern healthcare, such as the chronically ill. These typically take the form of reports that healthcare professionals do not listen to their concerns, or that their reportage about their medical condition is ignored or marginalized, or that they encounter substantive difficulties in their efforts to make themselves understood to the persons charged with their diagnosis and treatment.

The second are *physician complaints*, understood broadly as those offered by healthcare professionals. They often complain that patients provide medically irrelevant information, make odd statements and superfluous remarks about their condition, or otherwise fail to contribute epistemically to the collection of medical data. Taken together, a difficult epistemic situation emerges in which neither group can engage in effective testimonial and hermeneutical relations with the other.

Two features of these epistemic complaints are worth noting. The first is that they tend to have the consequence of complicating – and, in certain cases, compromising – the epistemic relationship between ill persons and the healthcare professionals charged with their care. Such breakdowns in relationship have a range of practical consequences, including the unwillingness or inability of ill persons to give complete or accurate reports of their symptoms and adherence to treatment, which in turn can create a need for additional tests or further referral. The practical consequences of such behaviours can include the jeopardising of the ill person's treatment and significant costs for the healthcare system when important information is overlooked. Moreover such breakdowns in the epistemic relationship can result in ill persons having negative subjective experiences of healthcare, such that they might come to associate hospitals not only with sickness and suffering, but also with confusion and isolation.

The second feature of these epistemic complaints is that they are systematic and longstanding features of healthcare systems, rather than isolated or incidental cases of communicative failure in otherwise epistemically harmonious systems. An abundant body of empirical evidence indicates that the epistemic complaints described are not anomalous but rather indicators of serious and persistent problems that arguably arise from contingent structural features of contemporary healthcare practice, but which could be addressed through systematic reform. In the late 1960s and early 1970s, paediatrician Barbara M. Korsch published an influential series of papers identifying ‘gaps in doctor‐patient communication’. Based on interviews with patients, she identified the causes of these ‘gaps’ being the ‘coldness’ associated with impersonal diagnostic procedures, doctors’ perceived indifference to or ignorance of the concerns of their patients, and a disorientating use of medical jargon.^3^

Korsch's work was followed by further studies of the personal and social experiences of ill persons, by authors such as Arthur Kleinman and S. Kay Toombs, and has also inspired recent work in phenomenological pathography.^4^ Such concerns have also directly informed subsequent legislative changes to healthcare policy, such as the NHS Patient Charter and the NHS constitution in the UK.^5^ But despite this greater awareness, patients continue to voice epistemic concerns, most obviously through the vast body of pathographic literature – including online patient fora, blogs, and narratives – which consistently attest to persistent experiences of feeling ignored, marginalized, or epistemically excluded by health professionals.^6^ The UK Patients’ Association, for instance, lists complaints about communication between patients and health professionals as a frequent complaint received by the association.^7^ Although much of this literature focuses upon the emotional distress that such negative experiences generate, we think that they are grounded in an epistemic dimension that has not, until now, been fully identified.

Contemporary commentators have connected these epistemic complaints with their fears about a wider ‘crisis’ in modern medicine and to related calls for a ‘humanistic turn’ in medical care.^8^ Often the crisis and the calls for reform play upon and invoke distinctively epistemological concerns about the capacity of physicians to initiate and sustain rich testimonial and hermeneutical relationships with patients. Indeed, many medical policymakers have begun to issue calls for a restructuring of medical training and practice whose explicit aim is to ameliorate the communicative problems that arise between patients and physicians.^9^

In 2012, Darrell G. Kirch, the president of the Association of American Medical Colleges, announced an ambitious reform of the Medical Career Aptitude Tests (MCAT). From 2015, American medical students are required to study ethics, philosophy, and cultural studies so that they can cultivate and refine their capacities to understand and engage with the concerns and experiences of their patients. As Kirch writes, ‘being a good physician is about more than scientific knowledge’, for it involves ‘understanding people’, thereby requiring that doctors have ‘communication skills, and an ability to interact with people with empathy [and] integrity’ – a clear testimony to the need to respond to the epistemic complaints noted above.^10^ It is therefore clear that the two sets of epistemic problems we identified reflect systematic features of modern healthcare that are of significant concern to patients, practitioners, and policymakers.

Other recent developments in attitudes to healthcare, mainly in the United States and United Kingdom, such as the growth of ‘narrative medicine’, are also interpretable as responses to those epistemic problems; however, typically the epistemological issues that generate the problems are neither recognised nor addressed. An example is the increasing emphasis placed upon ‘communication skills’ that doctors ought to exercise, which tend to focus on superficial modifications of physical behaviour – such as adopting an open posture and making eye contact. Such measures arguably fail to identify and challenge the deeper epistemic inequalities that characterise the relationship between patients and physicians. The use of reductive and simplistic questionnaires to glean information about ‘patient satisfaction’ is another such example, in which opinions are sought, but only in a confined and predefined form that is sensitive only to measurable and schematised variables of patient experience.

Although such skills, initiatives, and policies should be welcomed as efforts to repair the epistemic relationship between patients and physicians, they tend to rely upon highly contestable epistemological presuppositions. For instance, they tend to neglect the fact that those relationships are usually characterised by epistemic inequality and asymmetric power relations in a way that upsets comfortable hopes about the efficacy of superficial behavioural changes by physicians. Being physically or mentally unwell, dependent on others for pain relief, bedbound, or incontinent – all of these place the patient in a position of vulnerability and dependence which erodes their social and epistemic confidence and capacities.^11^ We claim that concerns about patient‐physician relationship, the efficacy of medical care, and public trust in medicine can be usefully reframed in terms of fundamental epistemological problems. If so, one should expect epistemology to play an essential role in the diagnosis and treatment of these problems.

The aim of this article is therefore to provide an epistemological analysis of these problems using Miranda Fricker's notion of ‘epistemic injustice’.^12^ We make two claims, the first being that chronically ill persons are especially vulnerable to epistemic injustice, owing to prevalent negative stereotypes of illness and certain structural features of contemporary healthcare practice.

The second claim is that the concept of epistemic injustice can therefore help to explain, at least to some degree, patients’ complaints about their healthcare experiences. Since ill persons already encounter a range of difficulties, offering new explanatory and ameliorative resources is an essential task, and one to which applied epistemology can contribute.

In addition, there are particular constraints that restrict patients’ ability to describe their illness experience, and hence to provide their own account of the clinical encounter. These will be discussed below as a form of hermeneutical injustice, and we have also discussed them elsewhere.^13^ Although this is not the focus of this article, we note that the two phenomena – epistemic injustice and communicative constraints – intersect in significant ways in the case of illness.

Before beginning the analysis two caveats are needed. First, our claim is not that ill persons are always epistemically reliable, for that is clearly not the case. Certain cases of illness – such as certain brain injuries, dementia, and psychosis – result in severe cognitive impairment that will necessarily erode a person's epistemic reliability. Our claim is that judgments about the epistemic credibility of ill persons are too often prejudicial and generated and sustained by negative stereotypes and structural features of healthcare practice. Many patients will not be as well placed as their doctors to understand certain aspects of their illness and treatment, but this neither requires nor justifies the further attribution to those patients of inferior epistemic status *tout court*.

Second, our focus in this article will primarily be on the chronically ill, and so our references to ‘the ill’ should be understood as referring to the fairly stable social group of persons with chronic somatic illnesses and their associated stigma. Our aim here is to secure the claim that this specific sub‐group of ill persons can and does suffer epistemic injustice. We leave questions about specific and complex cases – for example, about how our analysis might apply to mental illness, patients who are children, and acute illness – for further work.^14^

## 2 Epistemic Injustice, Prejudice and Stereotypes

The concept of ‘epistemic injustice’ was introduced by Miranda Fricker to refer to forms of injustice that are ‘a wrong done to someone specifically in their capacity as a knower’.^15^ Since the social and epistemic practices of giving information to others and interpreting our experiences is integral to our rationality, identity, agency and dignity, it is evident that injustice which harms our testimonial and hermeneutical capacities will be a source of deep harm. In her book, Fricker focuses on epistemic injustices that arise from cases of racial and sexual stereotyping and prejudice, such as cases where a speaker's testimony is accorded a lower degree of credibility owing to their being black or a woman, than they would if hearing the testimonies of a white male. Fricker goes on to claim that the damage resulting from epistemic injustice impacts upon a person's life as a whole, and so ramifies with more familiar forms of social injustice. If so, epistemic injustice is integrally related to social injustice.^16^ We suggest that there are distinctive features of the social group of ill persons that render them vulnerable to similar forms of negative prejudices and stereotypes, such that Fricker's account of epistemic injustice can also be applied to them.

There are several varieties of epistemic injustice, but for reasons of space and focus, our analysis focuses on the two identified by Fricker, namely *testimonial injustice* and *hermeneutical injustice*, which arise in the context of our testimonial and hermeneutical practices. We also discuss two specific forms of testimonial injustice, which Christopher Hookway dubs *participatory prejudice* and *informational prejudice*, each of which involve a person or group facing obstacles to their participation in collective epistemic activities.^17^ The relationship of these two prejudices to testimonial injustice is a matter of debate among virtue epistemologists, with Fricker herself arguing that testimonial injustice is ‘the most basic’ form of epistemic injustice, of which the two prejudices identified as Hookway are specifications.^18^ We agree, so the article starts with testimonial injustice, moves onto the two prejudices, before turning to hermeneutical injustice. In each case, we ask whether ill persons are especially vulnerable to that type of injustice, and if so, identify the stereotypes, structures, and practices that generate and sustain it.

Illness often leads to incapacitation, anxiety, and insecurity; these can either be met with empathy and compassion, or may trigger negative stereotyping. Instead of making an empirically and reflectively robust judgment on a case‐by‐case basis of the epistemic credibility of an ill person, health professionals (and others) may tacitly fall back upon such uncritically (and often unconsciously) adopted stereotypes, many of which incorporate negative epistemic prejudices.

Fricker emphasises that such stereotypes and prejudices typically operate ‘without any focused awareness’ and are ‘(typically) culpably resistant to the evidence’, and thus irrational.^19^ Many prevailing stereotypes of ill persons tend to connote incapacitation, disability, diminished agency, social vulnerability, psychological fragility, and bodily failure, among others.^20^ These stereotypes often portray illness in terms of moral or conative failure or social defect that may ‘contaminate its surroundings’^21^ and range from moralistic forms through to the ‘punitive and sentimental’ types described by Susan Sontag.^22^ A broad theme of these stereotypes is, as Arthur Frank remarks, that ‘the power of stigma has fed on seeing the body's condition as an expression of morality’,^23^ such that the fact of illness is a mark of moral, social, and epistemic failure.

The structures of healthcare institutions are underpinned by biomedical approaches that focus upon the biological rather than existential aspects of illness, and therefore lower the level of attention paid to the subjective experience of being ill. Healthcare provision is based upon principles of efficiency (and in some cases financial profit) and designed to suit the needs of health specialists rather than patients. In addition, time pressure, short consultations, and use of standardised protocols that leave little room for personal needs and values, are also core features of modern healthcare systems.

Finally, work is task‐based rather than patient‐focused, thereby closing down opportunities for establishing sustained contact between a particular health professional and an individual patient, and for the rich forms of communicative relationship that might facilitate the recognition and cultivation of patients’ ability to contribute to the epistemic aspects of their care. As we argue below, all of these can contribute in a myriad of ways to the gradual erosion of the epistemic confidence and capacities of the ill person.

## 3 Testimonial Injustice

Testimonial injustice occurs when prejudice causes a hearer to give a deflated level of credibility to the speakers’ word.^24^ Specifically, a *negative stereotype* introduces *prejudices* that cause a *credibility deficit* with the consequence that a person's capacity to act as a testifier – a reliable giver of information – is impaired, or in severe cases completely destroyed. Fricker emphasises that stereotypes and prejudices are neutral, partly because they can be benign and sometimes advantageous, and partly because they serve an essential pragmatic cognitive and social role. The concern about testimonial injustice arises when a person or group are subjected to a ‘negative identity‐prejudicial stereotype’ which involves a disparaging association between a social group and one or more attributes, and is closely connected to ethically objectionable investment such as disdain for the relevant social group.^25^

A typical case of testimonial injustice could involve negative stereotypes of a particular race or gender as suffering inferior cognitive capacity and a presumed predilection for deception, such that their epistemic credibility is automatically downgraded. Such injustices can be incidental, if the stereotype is weak or the prejudices susceptible to challenge, or they can be systematic, especially if, as in the case of racism and sexism, the stereotypes and prejudices are deeply entrenched in the social world. In the latter case, the social group may suffer a ‘tracker prejudice’, as the prejudices imposed by the negative stereotype tracks them through different domains of the social world.^26^

A person or social group suffers from testimonial injustice when a negative identity‐prejudicial stereotype lowers their credibility in one of two ways. The first is that the person or group suffers a loss of testimonial authority owing to a credibility deficit, especially in relation to other socially and epistemically dominant groups who might enjoy a corresponding credibility excess. The second is that the person or group will gradually lose their epistemic confidence as they endure the constant erosion of their credibility, which may, with time and repetition, crush their confidence in their epistemic capacities. A person or group suffering from such a situation will not expect what they say to be heard, and in time might not speak at all, as the constant assault upon their testimonial practices gradually undermines their epistemic and social confidence.^27^

The question is whether ill persons are especially vulnerable to testimonial injustice as Fricker describes it; our answer is that they are. For ill persons are often subjected to one or more of a range of negative stereotypes, which, though diverse, often include attributions which tend to undermine their epistemic competence and capacities. These include the stereotypical description of ill persons as cognitively impaired or emotionally compromised, owing either to their somatic condition or their psychological reactions to it (*mutatis mutandis* for psychiatric illness); or as existentially unstable, gripped by anxieties about mortality and morbidity such that they ‘cannot think straight’; or that they will be psychologically dominated by their illness in a way that warps their capacity to accurately describe and report their experiences (e.g. ‘the moaner’ or ‘the drama queen’ stereotype).

Such negative stereotypes will therefore tend to prejudicially deprive ill persons of the prerequisites of reliable epistemic conduct, such as cognitive capacity and psychological stability, and also attribute to them characteristics – like intensified emotionality – that are usually (and contentiously) supposed to be opposed to rationality and reliability.^28^ The consequence of this negative stereotyping is that patients’ testimonies are unjustly accorded lower degrees of epistemic credibility than they otherwise would. A further consequence is that the testimonies that would ordinarily indicate testimonial reliability are interpreted as irregular or atypical – that one ‘got them on a good day’, say – thereby further skewing the evaluative engagement of those ill persons.

Consider the following account:

I had an abnormal cervical smear, so was sent to the large city teaching hospital for a coloscopy. I changed into the usual ties‐up‐the‐back gown, with the usual vital ties missing, and then went through for the examination. It's a bit uncomfy but I was ok. Lots of big sighs from the consultant with his head between my legs. Then off he goes, leaving the room. I'm told to follow. So I arrive, naked under a gown which doesn't do up, slightly damp between the legs and a bit stressed as I have to sit down and I'm worried about leaving a wet patch. He goes on to tell me I need an operation. I hear blah‐blah‐blah as I'm perching and panicky. And it's very difficult to think without your pants on. I said nothing.^29^

More generally one can identify features of contemporary healthcare practice that can encourage and entrench the testimonial injustice that ill persons experience. Working under constant time pressure, routinisation of tasks, and shift work all undermine opportunities to listen at length to what patients say and to create a relationship with them. Excessive formality of medical discourse, even when conversing with patients, and emphasis on professionalization hamper human interaction and may limit ways of listening. Indeed, the deleterious implications of these features have been recognised and discussed in literature on the design and reform of healthcare services, evident for instance in the great emphasis now placed on the solicitation and inclusion of ‘patient perspectives’.^30^

Yet both formal surveys and anecdotal evidence suggests that patient complaints persist and levels of dissatisfaction remain considerable.^31^ A constant feature of those complaints is the failure of health professionals to evince desired testimonial sensibilities, and their consequently being perceived by patients as cold, impersonal, or dismissive. Ill persons are therefore demonstrably vulnerable to testimonial injustice because entrenched features of the social and medical world incorporate a variety of negative stereotypes of ill persons that sustain prejudices that impose credibility deficits.

If ill persons are vulnerable to testimonial injustice, as we argue, there is still the question of the nature of the primary epistemic harm they suffer. Fricker characterises that harm in terms of objectification, but more recently Gaile Pohlhaus has suggested that it is more fully understood in terms of truncated subjectivity.^32^ Pohlhaus argued that the ‘primary harm of testimonial injustice’ should be defined as ‘being relegated to the role of epistemic other, being treated as though the range of one's subject capacities is merely derivative of another's’. More fully,

…unlike a subject (and more like an object), she is not seen as capable of contributing to epistemic practices *uniquely*, that is, from her own distinct lived experience in the world. Consequently, her epistemic labor contributes to the community via which epistemic interests are pursued, but she is not permitted to contribute in ways that would redirect epistemic practices toward those parts of her experienced world that extend beyond or trouble the veracity of the dominantly experienced world. Any contribution that might do so is summarily denied epistemic support and uptake by dominant members of the community.^33^

This seems right to us. Patients seem to occupy the position of ‘other’ in Pohlhaus’ sense, in that their testimony is regularly solicited and indeed essential (e.g. in reporting symptoms or side effects of a drug), but nonetheless they remain a ‘derivatised subject’, i.e. a subject whose capacities are reduced to attending only to what stems from the other's perspective.^34^ Patient testimonies are sought as sources of factual information, but testimony about the lived experience of illness and the clinical encounter, which may challenge the medical view, is often excluded from consideration and plays no formal role in decision‐making and service design (at least until very recently). Thus, in cases of testimonial injustice, patients are perceived as ‘somewhere between an epistemic subject and object’.^35^ This intersects with other types of objectification patients encounter in a medical setting, for example, the objectification of their bodies.^36^

Pohlhaus’ account accurately captures the epistemic plight of many ill persons in healthcare contexts: first, they are afforded limited capacities to epistemically contribute, usually by providing factual information, but not by offering their distinctive lived experiences. Second, their epistemic labour contributes to the epistemic practices of healthcare systems but they are not permitted to contribute in ways that redirect the interests or concerns of those systems. These two points can only be uncovered by an epistemic analysis; this shows both the theoretical efficacy and practical significance of such an analysis, which can open the way to reforming healthcare practices. The epistemic analysis can also be usefully connected with philosophical work in the phenomenology of illness, which aims to enable patients to order, discern and describe the experience of illness, which is often confusing, painful and difficult to communicate.^37^ Both empowering patients’ epistemic practices and tackling epistemic injustice by combating negative stereotypes are required.

## 4 Participatory and Informational Prejudice

Testimonial injustice occurs in cases of *prejudicial credibility deficit*. These can occur *during* our social‐epistemic interactions with others, as in the cases that Fricker focuses upon in her book. But, as Christopher Hookway observes, such deficits can actually begin even before one gets as far as epistemic interaction with others. Specifically, he identifies two types of prejudice that can impair a person's ability to engage in shared epistemic activities. Since any such activities will involve the (attempted) giving of information, we agree with Fricker that they ought to be considered as specific forms of testimonial injustice.^38^

The starting point for these prejudices is the fact that our participation in shared epistemic practices, such as debating or enquiring, is necessarily premised upon certain presuppositions about our epistemic peers. These presuppositions take the form of implicit or explicit expectations about the sorts of capacities and dispositions that our epistemic peers should typically exhibit. Hookway focuses on two presuppositions. First, that participants will have a *sense of relevance*, a capacity to determine which ideas are worth taking seriously, which objections are meritorious, and so on. A person who lacks a sense of relevance is likely to undermine the efficiency of any epistemic community they participate in by failing to properly judge the relevance of both their own contributions and those of others. Second, that our participants have a *capacity to provide information* that can meaningfully contribute to the epistemic task at hand, for instance to provide factual corroboration or correction, or to have sufficient background knowledge of the subject being debated. Hookway identifies two forms of epistemic prejudice attached to these two presuppositions which, when grounded in negative prejudicial stereotypes, have as their consequence the exclusion of certain persons and groups from participation in shared epistemic activities.

The first of these is *participatory prejudice*, which occurs when a person or group is prejudicially judged to lack capacities required for having a *sense of relevance*, and hence as not being suitable participants for collective epistemic activity. Hookway offers the example of the epistemic practice of *questioning*: when a person offers a question for consideration it must be appraised and this appraisal will affect the epistemic career of that question. A question that is positively appraised may be taken as relevant and insightful, to be taken seriously and therefore to be accepted as a basis for further discussion and action. By contrast, a question may be negatively appraised, perhaps by being judged to be irrelevant or ill‐formed, and so ignored and derogated and thus excluded from further epistemic consideration. The consequence of a negative appraisal is not only that the *question* is not taken up as a basis for further epistemic activity, but also that the *questioner* and the specific concerns, interests, and standpoints that motivate the question are likewise excluded. A question is, after all, typically asked because the questioner wants to raise awareness of certain issues, affect the agenda of a discussion, or correct the obliviousness of their peers to certain standpoints.

Certain negative prejudices can therefore manifest as participatory prejudices because their social and epistemic consequence is that one is prevented from ‘recognising [a potential participant] in debate or discussion’.^39^ In such cases, a well‐placed concern with epistemic efficiency is compromised and corrupted by being placed in the service of an epistemic prejudice, which is often unconscious.

We think that ill persons are vulnerable to participatory prejudice for at least two related reasons. The first is that ill persons may be supposed to lack the training and experience needed for the possession of a robust sense of relevance required if one is to make meaningful contributions to the epistemic practices of medicine. Since ill persons generally lack medical training they may be judged to lack the prerequisites for a sense of relevance in medical contexts. This judgment relies upon the implicit co‐definition of a sense of relevance with medical expertise, but this structurally excludes the overwhelming majority of ill persons from potential possession of a sense of relevance. If so, then this form of participatory prejudice would be a structural feature of contemporary healthcare practice. Moreover, what is intensely relevant to the ill person may be medically minor (e.g. incontinence) and vice versa, further adding to the sense of the irrelevance of the patient contributions.

The second reason ill persons are vulnerable to participatory prejudice is that they are typically regarded as the objects of the epistemic practices of medicine rather than as participants in them. For instance, a patient's participation may be confined to confirming basic biographical details or reporting symptoms. If one's stance towards a certain social group incorporates the perception of them as providers of information in this minimal sense, then it will be difficult for one to regard them as participants in a more epistemically substantive sense.

The second prejudice that Hookway identifies is *informational prejudice*. It occurs when a person or group is prejudicially judged to lack the ability to provide information relevant in a given context and hence as being an unsuitable participant in collective epistemic activity. This sort of prejudice can arise in two mutually reinforcing ways. First, it may appear as a *refusal to concede* the relevance or significance of the information being offered by a particular individual or group. This is especially liable to occur in cases where the type of information in question is not reflected in or recognised by the experience of the dominant social group. An epistemically dominant group may therefore refuse to concede that certain forms of information (such as qualitative reports of illness experiences) are relevant and significant to the epistemic task at hand, and therefore prevent it from informing the established epistemic practices; this also rules out the possibility of that information prompting *reform* of those practices.

Second, informational prejudice may appear as a *refusal to consider* presuppositions about the significance and types of information that are legitimate and admissible. An epistemic community will typically operate with a range of informational presuppositions for legitimate practical and cognitive reasons. Informational prejudice can arise when the members of that community refuse to periodically reconsider those presuppositions, especially in the face of vigorous and sustained calls by groups who argue for the significance of other forms of information. The decision to reassess those informational presuppositions is difficult to make and to follow through, due partly to the difficulties facing reform of foundational commitments. Hookway argues that because the excluded forms of information do not inform current practice it will generally be the case that an informational lacuna can only be detected from the ‘participant perspective’.^40^ This reflects the broader fact that the absence of social and epistemic resources can often only be identified and appreciated by those marginalized individuals and communities who suffer from their absence – a core sentiment of standpoint epistemology.^41^

We think that ill persons are vulnerable to informational prejudice in the two ways described. Ill persons are subject to refusals to concede the relevance of the types of information that they typically offer, such as information concerning their sense of bodily estrangement or worries about social isolation. Such information is not widely integrated into modern healthcare practice, and when it is, it tends to be classified as ‘subjective’, ‘non‐medical’, and often does not trigger action.

Ill persons are also subject to refusals to consider the possible significance of the forms of information that are being excluded, for instance by accepting that existential changes that accompany experiences of illness are important. It is well documented that despite persistent and often poignant testimonies from ill persons, psychologists, philosophers, and phenomenologists, the epistemic norms of medicine remain focused on quantitative and ‘objective’ forms of information.^42^

The persistence of structural informational prejudice also has important implications for the possibilities of reforming modern healthcare practices and institutions to address the epistemic problems described here. Efforts to design medical interventions without appropriate consultation with patients may result in healthcare systems that are not only epistemically unjust but also practically inefficient because a rich range of forms of information required for the genuine improvement of services is structurally excluded. We therefore suggest that the achievement of epistemically just healthcare practices and institutions requires the revision of underlying epistemic presuppositions and a strong epistemic pluralism that incorporates the informational contributions of patients.

Participatory and informational prejudices are related to and exacerbated by many of the social and historical factors that shape modern healthcare systems. Many of these factors have already been discussed – including the professionalization of medicine and the prevalent negative stereotypes of ill persons – but others present themselves. These include the practical pressures upon healthcare practitioners to meet targets and performance indicators, which impose strict limits upon the duration and type of informational exchanges between patients and physicians. Another is the typical socialisation of health professionals, in particular physicians, which encourages them to think of illness in purely physiological terms and to defend themselves against difficult emotions by emotionally distancing themselves from certain situations. And finally, the awareness that taking practical and policy measures to properly respond to informational and participatory prejudices would require radical changes in medical education and training, as illustrated by the scale of the MCAT reforms.

One might argue that the informational and participatory prejudices experienced by ill persons are perhaps grounded in a much broader tendency within the history of Western culture and philosophy to locate epistemic authority in persons who are *healthy* – as well as white, male, and adult – though there are a few honourable exceptions, including Friedrich Nietzsche and Alasdair MacIntyre.^43^ In several later works, MacIntyre argued that Western ethical and political philosophies have tended to disregard the fact that the lives of human beings are generally marked by ‘affliction, vulnerability, and dependence’, with the consequence that our conceptions of the good life and of a just society are therefore designed with an idealised image of the moral agent – independent, rational and autonomous – which obtains only temporarily and incompletely, and only for some. MacIntyre therefore argued that ethical and political thought ought to be reformed to reflect the fact that most persons are, in his phrase, ‘dependent rational animals’.^44^

We suggest that epistemology would also benefit from a greater recognition of the fact that the lives of epistemic agents are also characterised by affliction, vulnerability, and dependence. Study of the epistemic injustices experienced by the chronically ill is a useful starting point. But such a study ought to focus not only upon the prejudicial credibility deficits that constitute testimonial injustice discussed so far, but also upon the second basic type of injustice identified by Fricker, to which we now turn.

## 5 Hermeneutical Injustice

Hermeneutical injustice occurs when a gap in collective hermeneutical resources puts a person or group at unfair disadvantage when making sense of their social experiences. Specifically, ‘some significant area of one's social experience [is] obscured from collective understanding owing to a structural identity prejudice in the collective hermeneutical resource’.^45^ Certain experiences cannot be understood because of the absence of the resources needed to understand them, and then to articulate and communicate that understanding to others, with the consequence that the person or group suffers *hermeneutical marginalization*, which Fricker defines as ‘unequal hermeneutical participation with respect to some significant area(s) of social experience’.^46^

The social implications of hermeneutical marginalization arise because our ability to recognise and respond to the concerns and experiences of others is crucially premised upon our ability to understand those concerns and experiences by mobilising adequate hermeneutical resources. If the required resources do not exist, those experiences will remain obscure in a way that prevents even the most sympathetic social peers to respond adequately to them. It is for this reason that hermeneutical injustice is structural and not agential, arising from specific contingent features of the social world – such as its educational practices and political arrangements – rather than from the activities of specific agents. Indeed, a consequence of hermeneutical injustice is what Fricker calls *cognitive disablement*. All members of a society that is structurally hermeneutically unjust will be impaired in their capacity to understand certain experiences, but the impairment has differential impact.

Fricker offers the example of societies that lack the concept of sexual harassment, noting that though both the harasser and harassee are cognitively disabled, there is an obvious sense in which this is to the harasser's advantage: ‘it suits his immediate purpose, in that it leaves his conduct unchallenged’.^47^ Indeed, many cases can be offered in which a particular social group actively benefits from their inability to understand, and hence take seriously, the experiences of other groups. Bacon's maxim that knowledge is power is true, but in certain contexts ignorance can also be a source and means of legitimating power.^48^

Hermeneutical injustice can arise in at least three ways, the first two of which are identified by Rebecca Mason.^49^ She argues that a distinction should be drawn between *dominant* and *non‐dominant* hermeneutical resources on the grounds that ‘marginalized groups can be silenced relative to dominant discourses without being prevented from understanding or expressing their own social experiences’.^50^ The first way that hermeneutical injustice can arise is a situation characterised by a *global lack* of hermeneutical resources: the required hermeneutical resources do not exist and so are not available to any person or group within that society, including but not limited to the specific social group whose experiences they are. The second way is that a particular social group has perfectly adequate hermeneutical resources of its own – its members can make sense of their experiences – but those resources are not recognised or respected by the dominant social and epistemic authorities. This group therefore has non‐dominant hermeneutical resources, and so suffers hermeneutical injustice because the dominant social groups cannot understand their experiences.

We add to this a third way in which hermeneutical injustice can arise, through what we call *epistemic isolation*: situations where a person or group lacks the knowledge of, or means of access to, particular information; for instance, if they live within a politically repressive society which forbids access to the necessary sources of information in order to protect the government's hegemony (for example, by blocking certain websites, outlawing certain literature, and so on).^51^ We suggest that ill persons have non‐dominant hermeneutical resources and so suffer from hermeneutic injustice for that reason; i.e. ill persons can make sense of their experience but these lack the social recognition and epistemic respect in ways that are detailed below. We suggest that ill persons are less prone to the first and third modes of hermeneutical injustice, although it remains to be seen in further work whether some areas of illness are subject to a global lack of hermeneutical resources.

In the case of illness, hermeneutical injustice arises because the resources required for the understanding of the social experiences of ill persons are not accepted as part of the dominant hermeneutical resources. Most ill persons are capable of describing their experiences in non‐expert terms, but such experiences are: a. largely considered inappropriate for public discussion and b. play little or no role in clinical decision‐making. Such experiences are often considered private, if not shameful, and inappropriate for sharing with others. They are also stigmatising (cf. Goffman) and therefore talking about them can exact a social and personal cost from the ill person (e.g. disclosing one's HIV status can lead to social exclusion in certain groups).^52^

Ill persons’ accounts are often dismissed as ‘moaning’ or as idiosyncratic experiences shaped by the negative attitudes of the ill person (who is a ‘moaner’ or ‘expects too much’). And unless subsumed under a formal complaints procedure, such experiences remain largely un‐noted by healthcare providers. Such experiences play little role in the design of clinical services, regulation of service performance, or in the production of clinical guidelines. The inherently qualitative nature of such accounts makes them difficult to quantify and they are hence considered inadmissible evidence by medical decision‐making bodies.^53^ Recent attempts in the UK to shoehorn the diversity of illness experiences into the ‘friends and family test’ further reduce the richness of such experiences to a simple binary question.^54^

Hermeneutical injustice can arise through two sorts of strategies, defined here as stable social and epistemic practices which act to prevent the promotion of non‐dominant hermeneutical resources. The first are *strategies of exclusion*, which take the form of excluding a currently hermeneutically marginalized group from the practices and places where social meanings are made and legitimated, such as professional committees or legislative bodies. Such exclusion can take different forms, from physical exclusion to subtler forms of epistemic exclusion, such as the procedural insistence upon the employment of strenuous legal, medical, or academic terminologies and conventions, so as to exclude those who are not members of those groups from participating in deliberative processes.

The second are *strategies of expression*, in which a social group is excluded because, as Fricker argues, its ‘characteristic expressive style [is not] recognized as rational and contextually appropriate’; if, for instance, adopting an ‘intuitive or an emotional expressive style means that one cannot be heard as fully rational’.^55^ If so, the style that a marginalized group uses in its efforts to make the case for the recognition of its hermeneutical resources can serve to undermine those very efforts. And this can lead to a vicious circle of increasing frustration, leading to more extreme styles of expression, which in turn lead to further epistemic disenfranchisement. Those two strategies can, of course, operate simultaneously, pulling in different directions, especially since hermeneutical marginalization is often allied to wider forms of social exclusion.

The question, then, is whether ill persons are especially vulnerable to hermeneutical injustice in the sense offered by Fricker. Our suggestion is that they are: ill persons typically have non‐dominant hermeneutical resources that are not recognised or respected by the epistemically dominant healthcare professions, but which are essential to the understanding of at least certain aspects of the experience of illness. Ill persons can be, and often are, victims of strategies of exclusion: they often report that they are forced to adopt an epistemically marginal role in consultative exercises, or that they are required to employ the language and conventions which require professional training and experience, or that their preferred expressive styles are implicitly and derogatively interpreted as irrational (e.g. being labelled a ‘difficult patient’).

Such pejorative judgments of those expressive styles is, in fact, reflective of a wider philosophical prejudice that contrasts rationality and emotionality, thereby encouraging a sense that reason ought to be expressed coolly and dispassionately.^56^ This prejudice is especially apposite to cases of illness because these will typically have an intense and dramatic impact upon a person's life and so naturally invite expression in emotive and anecdotal styles. If so, a person operating with the rationalistic prejudice will unavoidably find it difficult to regard the expressive styles that ill persons find both natural and appropriate as properly rational.

These expressive styles may be overtly emotional and moreover display emotions that are socially unaccepted such as anger and envy. The expression may be nonlinear, jumping between different events and times, and hence confusing. It may be repetitive, as part of a process of acceptance. Or the expression may centre on themes that are difficult to accept, such as mortality, pain, and isolation. The new areas of ‘arts and health’, medical humanities, and music and dance therapies, as well as the phenomenology of illness, have developed as a critical reaction to this set of prejudices, and aim to elucidate and legitimate varied forms of expression in order to validate illness experiences. They can be seen as attempts to combat hermeneutical injustice by providing expressive means to both make sense of and communicate the experience of illness.

The hermeneutical injustice that ill persons are particularly susceptible to generally takes the form of a double injury, because the marginalization of their resources and expressive styles exacerbates the already considerable hermeneutical challenges that ill persons commonly face. Many experiences of illness tend to evince two hermeneutical features. The first is *inarticulacy*, the difficulty of adequately communicating, sharing, or ‘getting across’ certain aspects of the experience of illness.^57^ That is one reason why ill persons often engage in a search to identify or create novel communicative resources, such as visual art, film, music, poetry, or drama. And this is another reason for the development of the disciplines and therapies described above.

The second is *ineffability*, the sense that certain aspects of those experiences cannot be adequately communicated to others through propositional articulation at all because understanding is premised upon a person's having had the requisite bodily experiences.^58^ Typical examples of such ineffable experiences that are hermeneutically accessible only to those who have had the corresponding personal experiences might include childbirth, participation in violent military conflict, extreme or chronic pain, or ecstatic religious experience.^59^

Such experiences of inarticulacy and ineffability are sources of hermeneutical frustration and the development of artistic and other forms of expression can therefore be understood as attempts to expand and enrich the hermeneutical resources available to people who have been through such an experience. These efforts and the academic work carried out under medical humanities, narrative medicine and phenomenology of illness have made a considerable contribution to the cultural and social legitimacy of illness experiences, but remain marginal to medical understandings of the corresponding diseases.^60^

Certain cases of hermeneutical injustice in the context of illness might also take the form of a self‐fulfilling prophecy. The hermeneutical frustration that ill persons experience might result in their becoming increasingly emotive, fraught, and tense, such that they in fact become epistemically unreliable, thereby gradually fulfilling the prejudices inherent in the negative stereotypes that created the initial hermeneutical difficulties. Such cases would see the establishment of self‐sustaining gaps in collective hermeneutical resources by depriving those whom they marginalize and impair of the confidence and capacities to challenge and correct them.

## 6 Conclusions

This article provided an analysis of the epistemic problems prevalent in modern healthcare practice by using Fricker's notion of epistemic injustice. We argued that patient and health professionals’ epistemic complaints can be understood in terms of one or both of the two basic types of epistemic injustice: testimonial and hermeneutical injustice, where the former can manifest in the specific forms of participatory and informational prejudice. Such injustices are generated and sustained by both negative stereotypes of ill persons and structural features of modern healthcare practice. It is plausible to propose that at least certain specific sub‐groups of ill persons are victims of what Fricker calls ‘persistent and systematic epistemic injustice’.^61^

Our claim is not simply that patients have the *subjective* sense that their epistemic offerings are rejected, but that there is a systematic overlooking of the realm in which patient testimonies and interpretations carry great epistemic authority, namely, the lived experience of illness. The simplistic notion that patients all want the same thing (to get better) and that objective health states correlate well with subjective wellbeing leads to the epistemic effacing of this realm. Recent literature in health economics, happiness studies, narrative medicine and health psychology, clearly demonstrates that patients want many different things and that the relationship between objective health and subjective wellbeing is complex and ill‐understood.^62^

By opening an epistemic space for the lived experience of illness, an important new domain of knowledge – and of further epistemic enquiry and changing of the epistemic balance between patients and health professionals – can be revealed. This will also supplement the phenomenological study of the experience of illness by articulating the epistemic dimension of this study, something that has not been done to date. We hope to do this in a future paper.

Our analysis also offers several further potential avenues for future enquiry that could both refine our diagnoses of the sources of those epistemic injustices and indicate means for their resolution, of which five are noted here. First, it is important to uncover epistemic injustice in illness because, as with other forms of injustice, recognition and acknowledgment are the essential first steps towards resolution and abolition – difficult as those may be.

The second is to undertake critical analysis of the range of stereotypes of ill persons and to identify how distinctive stereotypes engender different forms of epistemic injustice, especially since it seems clear that there is a complex plurality of ‘stereotypes of the sick’.^63^

The third is to begin a social‐epistemic study of the ways in which epistemic injustices against ill persons are generated and perpetrated, and which might focus upon a systematic study of the strategies of exclusion and of expression and of the practices by which the epistemological presuppositions of medical practice are established. Such a study could begin by considering existing critiques of healthcare with an overt epistemic character: an obvious candidate is the concept of ‘medicalization’, whose relationship to epistemic injustice is, as Alistair Wardrobe argues, both nuanced and informative.^64^

The fourth is the application of a ‘phenomenological toolkit’ which draws upon the resources of existential phenomenology to provide ill persons with the means of overcoming the inarticulacy and ineffability of their experiences of illness and healthcare practitioners with the hermeneutical resources required for better understanding those testimonies.^65^ In a recent paper, Lauren Freeman argues that pregnant women suffer from epistemic injustice because their epistemically privileged perspective on their embodied situation is neglected within current healthcare systems. Freeman concludes that a phenomenological sensitivity to pregnant women's lived experience can therefore challenge the epistemic injustices that, she argues, those women suffer.^66^

The fifth would be to ask how, if at all, the structures and institutions of contemporary healthcare could be reformed in a way that would minimise their disposition to generate epistemic injustice, building upon a handful of recent studies exploring the relationship between epistemic justice and institutions.^67^

These various avenues for enquiry all indicate not only that epistemologists can make contributions to the study and resolution of the epistemic problems that are of such concern to patients, health professionals, and policymakers, but also that they can correspondingly benefit from engaging with social and institutional epistemic practices with such an obvious capacity to help or hinder human wellbeing.

Epistemologists will find rich examples of the complex epistemic situations and relations that obtain between particular social groups and structures, as well as new opportunities to understand how certain virtues, such as epistemic justice, might play a distinctive role in the healthcare professions. We hope this article will provide both direction and inspiration for further studies of the epistemic injustices that affect ill persons.

## Notes

1 Rita Charon, *Narrative Medicine: Honoring the Stories of Illness* (New York: Oxford University Press, 2008), p. 66.

2 H.B. Beckman & R.M. Frankel, ‘The effect of physician behavior on the collection of data’, *Annals of Internal Medicine* 101 (1984): 692–696.

3 Barbara Korsch *et al*., ‘Gaps in doctor‐patient communication: Doctor‐patient interaction and patient satisfaction’, *Pediatrics* 42 (1968): 855–871, and ‘Gaps in doctor‐patient communication II: Patients’ response to medical advice’, *The New England Journal of Medicine* 280 (1969): 535–540.

4 See Arthur Kleinman, *Patients and Healers in the Context of Culture: An Exploration of the Borderland between Anthropology, Medicine, and Psychiatry* (Berkeley, CA: University of California Press, 1980); and *The Illness Narratives: Suffering, Healing, and the Human Condition* (New York: Basic Books, 1988); S. Kay Toombs, *The Meaning of Illness: A Phenomenological Account of the Different Perspectives of Physician and Patient* (Dordrecht: Kluwer, 1993); Havi Carel, *Illness: The Cry of the Flesh* (London: Routledge, 2013).

5 For the NHS Constitution see (accessed 15 July 2013): http://www.nhs.uk/choiceintheNHS/Rightsandpledges/NHSConstitution/Documents/2013/the-nhs-constitution-for-england-2013.pdf; for the Patient Charter (accessed 30 September 2015) see: Department of Health (1991) *The Patient's Charter* (London: HMSO). The charter was first published in 1991 and revised in 1995 and 1997. See also Greg Dyke, *The New NHS Charter: A Different Approach* (London: Department of Health, 1998).

6 A classic source is Arthur Frank, *The Wounded Storyteller: Body, Illness, and Ethics* (Chicago, IL: University of Chicago Press, 2010). Chapter seven is titled ‘Testimony’.

7 See http://www.patients-association.org.uk/helpline/common-complaints/ (accessed 29 September 2015).

8 See, for instance, Donald Marcum, *An Introductory Philosophy of Medicine: Humanising Modern Medicine* (Dordrecht: Springer, 2008).

9 See Gwen Marram van Servellen, *Communication Skills for the Healthcare Professional: Concepts, Practice, and Evidence*, 2nd edn. (London: Jones and Bartlett, 2009).

10 Darrell G. Kirch, ‘MCAT 2015: An open letter to pre‐med students’, March 2012, available at https://www.aamc.org/newsroom/reporter/march2012/276772/word.html (accessed 30 September 2015).

11 Derek Sellman, ‘Towards an understanding of nursing as a response to human vulnerability’, *Nursing Philosophy* 6,1 (2005): 2–10. On the ways in which ethics should be rethought in light of human vulnerability, see Alasdair MacIntyre, *Dependent Rational Animals* (London: Duckworth, 1999).

12 Miranda Fricker, *Epistemic Injustice: Power and the Ethics of Knowing* (Oxford: Oxford University Press, 2007).

13 Havi Carel & Ian James Kidd, ‘Epistemic injustice in healthcare: A philosophical analysis’, *Medicine, Healthcare, and Philosophy* 17,4 (2014): 529–540.

14 For an analysis of epistemic injustice in the case of children patients see Havi Carel & Gita Gyorffy, ‘Illness, epistemic injustice and paediatrics: The case of children’, *The Lancet* 384,9950 (2014): 1256–1257.

15 Fricker 2007 op. cit., p. 1.

16 Miranda Fricker, ‘Epistemic justice as a condition of political freedom’, *Synthese* 190 (2013): 1317–1332.

17 Christopher Hookway, ‘Some varieties of epistemic injustice’, *Episteme* 7 (2010): 151–163.

18 Miranda Fricker, ‘Replies to Alcoff, Goldberg, and Hookway’, *Episteme* 7 (2010): 164–178, especially pp. 174f.

19 Fricker 2007 op. cit., pp. 39 and 34.

20 For analyses of these stereotypes see Susan Sontag, *Illness as Metaphor and AIDS and its Metaphors* (Harmondsworth: Penguin Books, 1991); Arthur Frank, *At the Will of the Body* (New York: Mariner Books, 2002); Erving Goffman, *Stigma: Notes on the Management of a Spoiled Identity* (New York: Simon & Schuster, 1963). For studies focusing on strong negative reactions to illness by health professionals see D. Holmes; A. Perron & P. O'Byrne, ‘Understanding disgust in nursing: Abjection, self, and the other’, *Research and Theory for Nursing Practice* 20,4 (2006): 305–316 and D. Prior, A. Mitchell, M. Nebauer & M. Smith, ‘Oncology nurses’ experience of dimethyl sulfoxide odor’, *Cancer Nursing*, 23,2 (2000): 134–140.

21 Frank 2002 op. cit., p. 92.

22 Frank 2002 op. cit., p. 96; Sontag, *Illness as Metaphor*, p. 3; Barbara Ehrenreich. *Smile or Die; How Positive Thinking Fooled America and the World* (London: Granta Books, 2010).

23 Frank 2002 op. cit., p. 96.

24 Fricker 2007 op. cit., p. 17.

25 Fricker 2007 op. cit., p. 35.

26 Fricker 2007 op. cit., p. 27.

27 Fricker 2007 op. cit., pp. 47–48.

28 See, for instance, the essays in Part III of Peter Goldie (ed.), *The Oxford Handbook of the Philosophy of the Emotions* (Oxford: Oxford University Press, 2012).

29 This example is taken from responses to a query we posted on a patient mailing list in 2012.

30 See Angela Coulter & Jo Ellins, *Patient Focused Interventions: A Review of the Evidence* (London: The Health Foundation, 2009, available at www.health.org.uk); Ian Greener, *Healthcare in the UK: Understanding Continuity and Change* (Bristol: The Policy Press, 2009), chapter 8; Shirley McIver, ‘User perspectives and involvement’ in Kieran Walshe & Judith Smith (eds) *Healthcare Management*, 2nd edn. (Maidenhead: Open University Press, 2011), pp. 354–372.

31 For example, a 2014 King's Fund survey found a 65% satisfaction with the UK's National Health Service (http://www.kingsfund.org.uk/projects/bsa-survey-2014, accessed 30 September 2015). See also notes 4–7 above.

32 Gaile Pohlhaus, ‘Discerning the primary epistemic harm in cases of testimonial injustice’, *Social Epistemology* 28,2 (2014): 99–114.

33 Ibid., p. 107.

34 Ibid., p. 105.

35 Ibid., p. 107.

36 See Havi Carel & Jane Macnaughton. ““How do you feel?”: Oscillating perspectives in the clinic”. *The Lancet*, 23 June (2012): 2334–2335.

37 See, for instance, Carel *Illness* op. cit.; Havi Carel, ‘Phenomenology and its application in medicine’, *Theoretical Medicine and Bioethics* 32,1 (2011): 33–46; Havi Carel, *Phenomenology of Illness* (Oxford: Oxford University Press, forthcoming); Fredrik Svenaeus, ‘What is phenomenology of medicine? embodiment, illness, and being‐in‐the‐world’ in Havi Carel & Rachel Cooper (eds) *Health, Illness, and Disease* (Durham: Acumen, 2013); Frederik Svenaeus, ‘The phenomenology of health and illness’ in S. Kay Toombs (ed.) *Handbook of Phenomenology and Medicine* (Dordrecht: Kluwer, 2001, pp. 87–108; S. Kay Toombs, *The Meaning of Illness: A Phenomenological Account of the Different Perspectives of Physician and Patient*‬ (Dordrecht: Kluwer, 1993).‬‬

38 Fricker has recently developed the notion of ‘structural testimonial injustice’ to clarify the preemptive character of certain testimonial injustices. See (or, rather, hear) her keynote lecture, ‘Epistemic Injustice Revisited’, delivered at the conference *Understanding Epistemic Injustice*, University of Bristol, 26 June 2014, available to download at http://www.bristol.ac.uk/philosophy/research/epistemic-injustice-2014/

39 Hookway op. cit., p. 156.

40 Hookway op. cit., p. 158.

41 See Alison Wylie, ‘Why standpoint matters’ in S. Harding (ed.) *The Feminist Standpoint Reader: Intellectual and Political Controversies* (London: Routledge, 2004), pp. 339–351.

42 See Toombs 1993 op. cit.; Frank 2002 op. cit.

43 See, for instance, Genevieve Lloyd, *Feminism and History of Philosophy* (Oxford: Oxford University Press, 2002). Nietzsche's views on illness are discussed in Ian James Kidd, ‘Can illness be edifying?’, *Inquiry* 55,5 (2012): 496–520.

44 See MacIntyre op. cit., especially chapter 1.

45 Fricker 2007 op. cit., p. 155.

46 Fricker 2007 op. cit., p. 153.

47 Fricker 2007 op. cit., p. 151.

48 See Fricker 2007 op. cit., pp. 151 and 161–162f. See further the essays in Robert Proctor & Londa Schiebinger (eds), *Agnotology: The Making and Unmaking of Ignorance* (Stanford, CA: Stanford University Press, 2008).

49 Rebecca Mason, ‘Two kinds of unknowing’, *Hypatia* 26 (2011): 294–307.

50 Mason op. cit., p. 294.

51 See David Coady, *What To Believe Now: Applying Epistemology to Contemporary Issues* (Oxford: Wiley‐Blackwell, 2012), chapters 4–6.

52 Goffman op. cit.

53 Megan Wainright & Jane Macnaughton, ‘Is a qualitative perspective missing from COPD guidelines?’ *The Lancet Respiratory Medicine* 1,6 (2013): 441–442.

54 See http://www.england.nhs.uk/statistics/statistical-work-areas/friends-and-family-test/friends-and-family-test-data/ (accessed on 30 Sep. 15).

55 Fricker 2007 op. cit., pp. 160–161.

56 See Martha Nussbaum, *Love's Knowledge: Essays on Philosophy and Literature* (Oxford: Oxford University Press, 1990); Mikel Burley, ‘Emotion and anecdote in philosophical argument: The case of Havi Carel's *Illness*’, *Metaphilosophy* 42,1–2 (2011): 33–48.

57 See Carel *Illness* op. cit.

58 See Havi Carel, ‘Bodily doubt’, *Journal of Consciousness Studies* 20,7–8 (2013): 178–197.

59 See David Biro, ‘When language runs dry: Pain, the imagination and metaphor’ in L. Folkmarson Käll (ed.) *Dimensions of Pain* (London: Routledge 2013), pp. 13–26; Cressida J. Heyes ‘Child birth: An aesthetic’, in *Dimensions of Pain*, pp. 132–141; Mark Wynn, *Emotional Experiences and Religious Understanding: Integrating Perception, Conception, and Feeling* (Cambridge: Cambridge University Press, 2005).

60 A novel and energetic exception to this is the ‘Medicine Unboxed’ project (https://plus.google.com/+medicineunboxed, accessed on 30 September 2015).

61 Fricker 2007 op. cit., p. 58.

62 See E. Angner, N.R. Midege, K. G. Saag & J. Allison, ‘Health and happiness among older adults: A community‐based study’, *Journal of Health Psychology* 14,4 (2009): 503–512; J.C.J.M. de Haes & F.C.E. van Knippenberg, ‘The quality of life of cancer patients: A review of the literature’, *Social Science and Medicine* 20,8 (1985): 809–817; H.M. Johnson, ‘Unspeakable conversations’, *New York Times*, 16 February 2003. For an overview and philosophical analysis of the discrepancy between objective health and subjective wellbeing, see Havi Carel, ‘Ill, but well: A phenomenology of well‐being in chronic illness’ in J. Bickenbach, F. Felder & B Schmitz (eds) *Disability and the Good Life* (Cambridge: Cambridge University Press, 2014), pp. 243–270.

63 See for example Goffman op. cit.; Talcott Parsons, *The System of Modern Societies* (Englewood Cliffs, NJ: Prentice‐Hall, 1971). 

64 Alistair Wardrobe, ‘Medicalisation and epistemic injustice’, *Medicine, Healthcare, and Philosophy* 18,3 (2015): 341–352.

65 See Havi Carel, ‘Phenomenology as a resource for patients’, *Journal of Medicine and Philosophy* 37,2 (2012): 96–113; Carel & Kidd op. cit.; Matthew Ratcliffe, ‘Phenomenology as a form of empathy’, *Inquiry* 55 (2012): 473–495.

66 Lauren Freeman, ‘Confronting diminished epistemic privilege and epistemic injustice in pregnancy by challenging a “panoptics of the womb”’, *Journal of Medicine and Philosophy* 40,1 (2015): 44–68.

67 See Elizabeth Anderson, ‘Epistemic justice as a virtue of social institutions’, *Social Epistemology* 26 (2012): 163–173 and Miranda Fricker, ‘Are there institutional virtues?’ in Tamar Gendler & John Hawthorne (eds) *Oxford Readings in Epistemology*, vol. 3 (Oxford: Oxford University Press, 2009), pp. 235–252.

